# Androgen Regulated Genes in Human Prostate Xenografts in Mice: Relation to BPH and Prostate Cancer

**DOI:** 10.1371/journal.pone.0008384

**Published:** 2009-12-21

**Authors:** Harold D. Love, S. Erin Booton, Braden E. Boone, Joan P. Breyer, Tatsuki Koyama, Monica P. Revelo, Scott B. Shappell, Jeffrey R. Smith, Simon W. Hayward

**Affiliations:** 1 Department of Urologic Surgery, Vanderbilt University School of Medicine, Nashville, Tennessee, United States of America; 2 Dermatology Division, Vanderbilt University School of Medicine, Nashville, Tennessee, United States of America; 3 Vanderbilt Microarray Shared Resource, Vanderbilt University School of Medicine, Nashville, Tennessee, United States of America; 4 Department of Medicine, Vanderbilt University School of Medicine, Nashville, Tennessee, United States of America; 5 Department of Biostatistics, Vanderbilt University School of Medicine, Nashville, Tennessee, United States of America; 6 Department of Pathology, Vanderbilt University School of Medicine, Nashville, Tennessee, United States of America; 7 The Vanderbilt-Ingram Cancer Center, Vanderbilt University School of Medicine, Nashville, Tennessee, United States of America; 8 Department of Cancer Biology, Vanderbilt University School of Medicine, Nashville, Tennessee, United States of America; 9 Medical Research Service, VA Tennessee Valley Healthcare System, Nashville, Tennessee, United States of America; 10 Department of Pathology and Laboratory Medicine, University of Utah, Salt Lake City, Utah, United States of America; 11 Avero Diagnostics, Dallas, Texas, United States of America; Roswell Park Cancer Institute, United States of America

## Abstract

Benign prostatic hyperplasia (BPH) and prostate carcinoma (CaP) are linked to aging and the presence of androgens, suggesting that androgen regulated genes play a major role in these common diseases. Androgen regulation of prostate growth and development depends on the presence of intact epithelial-stromal interactions. Further, the prostatic stroma is implicated in BPH. This suggests that epithelial cell lines are inadequate to identify androgen regulated genes that could contribute to BPH and CaP and which could serve as potential clinical biomarkers. In this study, we used a human prostate xenograft model to define a profile of genes regulated *in vivo* by androgens, with an emphasis on identifying candidate biomarkers. Benign transition zone (TZ) human prostate tissue from radical prostatectomies was grafted to the sub-renal capsule site of intact or castrated male immunodeficient mice, followed by the removal or addition of androgens, respectively. Microarray analysis of RNA from these tissues was used to identify genes that were; 1) highly expressed in prostate, 2) had significant expression changes in response to androgens, and, 3) encode extracellular proteins. A total of 95 genes meeting these criteria were selected for analysis and validation of expression in patient prostate tissues using quantitative real-time PCR. Expression levels of these genes were measured in pooled RNAs from human prostate tissues with varying severity of BPH pathologic changes and CaP of varying Gleason score. A number of androgen regulated genes were identified. Additionally, a subset of these genes were over-expressed in RNA from clinical BPH tissues, and the levels of many were found to correlate with disease status. Our results demonstrate the feasibility, and some of the problems, of using a mouse xenograft model to characterize the androgen regulated expression profiles of intact human prostate tissues.

## Introduction

Benign prostatic hyperplasia (BPH) is extremely common in aging men, contributing to the pattern of morbidity known as lower urinary tract symptoms (LUTS) and resulting in significant annual healthcare costs [1]. Despite the availability of medical and surgical treatments for BPH there is still inadequate understanding of the processes involved in benign pathological growth of the human prostate *in vivo* [2]. Such information could serve to better predict which patients may benefit from current medical therapy or are more likely to progress to requiring surgical intervention, as well as inform the choice of new medical approaches targeting novel pathways.

BPH occurs as men age, and androgens are required for the development of the condition [3], [4]. BPH is characterized pathologically by glandular and stromal hyperplasia in the prostate transition zone (TZ) [5]. The reawakening of the embryonic inductive potential in the prostatic stroma has been proposed as a cause of BPH [3], [5], [6], [7]. This is based on the idea that prostate growth results from the local interplay of growth factors between the epithelial and stromal elements of the organ under the influence of testicular androgens, suggesting that androgen regulated genes play a major role in the disease. This hypothesis is supported by considerable experimental evidence in particular from tissue recombination models [8], [9], [10].

Prostatic inflammation has also been implicated in the pathogenesis of BPH [11], [12], [13], [14]. Inflammation is associated with the severity of BPH, and the MTOPS (Medical Therapy of Prostatic Symptoms) study suggests that the risk of BPH progression and acute urinary retention is greater in men with prostatic inflammation [13], [15]. Increased prostate inflammation may also result in the disruption of epithelial structure and architecture, resulting in increased serum levels of prostate specific antigen (PSA).

Prostatic growth, differentiation and adult function are dependent upon the presence of androgens. It is well established that androgens control growth and differentiation via mesenchymal-epithelial interactions. In the adult prostate, androgens are believed to act through the stromal androgen receptor (AR) to maintain a growth-quiescent gland [16], [17] and through the epithelial AR to elicit the secretory differentiated function of the prostatic epithelium [18]. In contrast to normal growth-quiescence, hyperplastic growth of glandular epithelium and stroma within the transition zone in BPH represents changes in the balance between cell division and death. BPH may thus inappropriately recapitulate key events in prostatic developmental biology. The mesenchymal and epithelial genes regulated by androgens that are important in abnormal prostate growth in BPH remain to be completely defined. Studies using high throughput cDNA microarrays with androgen stimulated carcinoma cell lines are unlikely to be relevant to either prostatic development or BPH. Transformed epithelial cells in culture have a markedly different phenotype, and associated proteome, from their *in vivo* counterparts. In addition such approaches do not allow for simultaneous detection of key stromal cell genes or for genes that may be modulated as a consequence of crucial epithelial-stromal interactions.

More biologically relevant studies utilizing cDNA microarray analysis of BPH tissues have been reported. Luo, et al. analyzed the expression of 6,500 human genes in prostate cancer and BPH tissues from patients undergoing radical prostatectomy or transurethral resection of the prostate (TURP) [19], [20]. A number of genes were found to be consistently upregulated in BPH compared to normal prostate. These included *IGF1*, *IGF2*, *TGFB3*, *BMP5*, *MMP2*, *COX2*, and *GSTM5*. However, these studies used tissues enriched for epithelial cells. As a result, the analyses could have missed potentially crucial genes expressed predominantly in stromal cells. These studies did not address whether any of the patients had undergone prior androgen-ablation therapy. Approaches based on RNA extracted from tissues also do not allow for direct manipulation of hormonal stimulation, limiting the ability to more directly detect genes regulated by androgens, which might serve as predictors of response to therapies targeting androgenic stimulation or as novel targets for alternate drug therapy in this androgen driven disease process.

In another relevant study, gene expression profiles in prostatic stromal cells from different prostate zones were analysed, comparing normal and diseased tissues [21]. A number of genes were found to be differentially expressed between BPH stroma and normal transition zone (TZ) stroma. While many potentially important stromal genes were identified that may play a role in BPH, these studies used isolated stromal cells cultured *in vitro*, which would likely result in altered gene expression patterns from those which would be seen *in vivo*.

In the present study we used oligonucleotide microarrays to examine the expression of androgen regulated genes in benign human prostate tissue growing as xenografts in severe combined immunodeficient (SCID) mice [22]. This approach maintains the biologically relevant interactions of epithelium and stroma as occurs *in vivo* and allows for direct manipulation of androgens, either by castration or hormonal supplementation of the host. The resulting data set was then analyzed to identify relatively highly expressed genes which were androgen regulated. In an effort to identify potential biomarkers amenable to future blood-based testing, emphasis was placed on genes whose products were known or predicted to be extracellular. Expression of these genes was then analyzed using qRT-PCR with cDNA pools derived from tissues from patients with minimal, mild, moderate and severe BPH pathology changes, or with CaP, to determine whether expression correlated with disease status.

## Materials and Methods

### Ethics Statement

De-identified human prostate tissue samples were obtained from the Vanderbilt Tissue Acquisition Core via the Department of Pathology in accordance with Vanderbilt IRB protocols. All patients signed informed consent approving the use of their tissues for unspecified research purposes. All experiments involving animals were conducted according to the Animal Welfare Act and approved by the Vanderbilt Institutional Animal Care and Use Committee. Animal care/welfare and veterinary oversight was provided by the Vanderbilt Divison of Animal Care.

### Histopathologic Analysis and Sample Selection

To determine the severity of BPH pathology changes for cases utilized for RNA extraction, TZ areas affected by glandular and stromal hyperplasia were outlined in whole mount sections from radical prostatectomy (RP) samples obtained via the Department of Pathology in accordance with Vanderbilt IRB protocols. TZ volumes were determined by planimetry with a digitized graphics tablet, in a manner identical to our routine determination of total tumor volume in RPs [23], [24], [25]. Preference was given to cases with small volume peripheral zone (PZ) tumors, so that any overall prostate enlargement will be due to TZ enlargement and to reduce the likelihood that hormonal metabolism potentially relevant to BPH would be altered by large volume prostate cancer [26]. BPH was categorized from 37 cases as minimal (min), mild, moderate, or severe, based on the following criteria. Min/control: TZ volume <4.5 cm^3^, prostate wt <34 g, No BPH nodules; Mild BPH: TZ volume 4.5–8.99 cm^3^ or prostate wt 34–44.99 g or BPH nodules; Moderate BPH: TZ volume 9–16.99 cm^3^ or prostate wt 45–59.99 g and BPH nodules; Severe BPH: TZ volume ≥17 cm^3^ or prostate wt ≥60 g and BPH nodules. Prostate cancer tissues were classified as moderately differentiated (Gleason scores 5–6) or poorly differentiated (Gleason scores 8–9). Snap frozen fresh tissue cores procured as described below were processed for RNA and histology [27].

### Sub-Renal Xenografting of Fresh Human TZ Samples and Hormonal Ablation Strategies

De-identified human prostate tissue samples were obtained from the Vanderbilt Tissue Acquisition Core via the Department of Pathology in accordance with Vanderbilt IRB protocols. 6 mm diameter cores obtained intraoperatively from fresh RP specimens were procured from the right and left TZ and PZ from mid to base and from mid to apex as described [27] and histologic analysis was performed on frozen sections of full thickness cross sections. Cores determined to contain normal TZ tissue were cut into pieces approximately 2–3 mm in thickness and 4–6 pieces were then xenografted beneath the renal capsules of adult male severe combined immunodeficient (SCID) mice [C.B-17/IcrHsd-scid mice (Harlan, Indianapolis, IN)]. TZ tissues from each of six patients were xenografted into sets of 10 castrated SCID mice with some groups of mice receiving tissue from two different patients when available, grafted onto contralateral kidneys. Five mice from each group were given sub-cutaneous implants consisting of a 2.5 cm length of silastic tubing (ID 1.98 mm x OD 3.18 mm, Dow Corning, Midland, MI) containing 25 mg of testosterone (PCCA, Houston, TX). The ends of the silastic tubing were sealed with Silicone type A medical adhesive (Dow Corning). A small quantity of corn oil was added prior to sealing the tubing to facilitate dissolution of the testosterone. After allowing the xenografts to establish for one month, the implants were removed from the testosterone supplemented mice, and 25 mg testosterone pellets (produced with a Parr Pellet Press, model 2816 with 4.5 mm die, Parr Instrument Company, Moline, IL) were implanted subcutaneously in the mice that had not received testosterone. Control mice were sacrificed at the time of androgen addition or removal, and the remaining mice from each group were sacrificed at 1, 3, 7, and 14 days following androgen addition or removal. Harvested xenografts were quickly dissected under magnification and snap frozen in liquid nitrogen. Frozen tissues were stored at −80°C.

### RNA Extraction and cDNA Microarrays

RNA extraction of snap-frozen TZ tissues and harvested xenografts was performed using a modification of previously described methods [27], [28]. Briefly, RNA was extracted using TRIzol (Life Technologies, Inc., Gaithersburg, MD), followed by a second RNA isolation using RNeasy (Qiagen Inc., Valencia, CA) with DNAse treatment. RNA samples were stored at −80°C. RNA quality was analyzed by the Vanderbilt Microarray Shared Resource (VMSR) using spectrophotometry (NanoDrop Technologies, Wilmington, DE) and bioanalysis (Agilent Technologies, Santa Clara, CA). RNA samples of xenografts were submitted to the VMSR for amplification (NuGen Systems, Inc., Traverse City, MI) and labeling, followed by hybridization to microarrays printed from the Human Release 2.0 OligoLibrary, (Compugen, San Jose, CA), containing 28,830 unique genes from a total of 29,134 oligos. The reference RNA was created by pooling RNA samples from both castrate and androgen treated tissues from day zero control mice.

### Statistical Analysis of Microarray Data

Due to the considerable variation inherent in individual patients, the time 0 for each tissue sample was used as the control or reference sample rather than a standard reference sample across the entire experiment. Data were normalized by Lowess using GeneTraffic software (Iobion Informatics, La Hoya, CA) and imported into GeneSpring (Agilent Technologies, Santa Clara, CA) for subsequent analysis. Initially, only day 0 and day 14 time points were considered, with genes that had at least a 1.5-fold upregulation in the day 14 sample vs. day 0 selected, which yielded 5,679 genes/probes. An ANOVA analysis was used to identify genes with significant differences in expression, with a *P*-value cut-off of 0.05, indicating 284 expected false positive genes. A Welch t-test was then used to identify genes with significant differences (*P*-value less than 0.05) in expression between days 0 and 14 across all tissue samples. This list was then filtered by signal expression value, retaining the top 95% of signal dynamic range, yielding 784 genes/probes. This method of analysis was independently performed for both the castrate and testosterone supplemented data sets. All statistical analyses of microarray data were performed by the VMSR.

### Identification of Candidate Biomarker Targets

Potential candidate genes were systematically selected with overlapping bioinformatics criteria, employing WebGestalt: 1) androgen-regulated (1.5-fold or *P*≤0.05 within our microarray data), 2) expressed at significantly higher levels in the prostate relative to other tissues (*P*≤0.05 by hypergeometric test), and 3) extracellular space or cell surface (Gene Ontogeny categories). These criteria yielded a set of genes that included previously validated biomarkers, including *KLK3*, *ACPP*, and *MSMB*. Several additional genes known or suspected to be involved in BPH based upon prior studies were “manually” included for further study: *IGF1*, *IGF1R*, *TGFB1*, *TGFB3*, *TGFBR1*, and *TGFBR2*. A total of 84 gene targets (and the *18S* rRNA housekeeping control gene) were chosen for confirmation of expression in patient samples, several assessed with redundant probes.

### Real-Time qRT-PCR Confirmation

A TaqMan low density microfluidic array card, format 96a (Applied Biosystems, Foster City, CA) was designed to assay candidate genes from the microarray analysis and control genes, for a total of 96 targets. 1 µg of total RNA was reverse transcribed into single-stranded cDNA using High-Capacity cDNA Archive kit (Applied Biosystems). Following cDNA synthesis, RNA was degraded by alkaline hydrolysis, adjusted to neutral pH, and cDNA purified by adsorption to silica gel (QIAquick PCR Purification kit, Qiagen Inc.) and eluted in 64 µl of 10 mmol/L Tris HCl (pH 8.5). cDNA quantities were measured spectrophotometrically (NanoDrop ND-1000, NanoDrop Technologies). cDNA was diluted to 0.25 ng/µl in 1X TaqMan Universal PCR Master Mix (Applied Biosystems), loaded into the microfluidic card, sealed and centrifuged. Cards were then cycled on an ABI Prism 7900HT sequence detection system, and data analyzed with SDS 2.1 software (Applied Biosystems). After normalization to the endogenous control, 18S rRNA, levels were expressed relative to the control RNA pool calibrator (fold change). RNAs prepared from each category of BPH tissues were pooled from 5–10 patients. Control RNA was pooled from patients with no appreciable TZ expansion. The analysis included four replicates for the mild and severe BPH pools, as well as the moderately differentiated and poorly differentiated prostate cancer pools, and eight replicates for the control and moderate BPH pools.

### Immunohistochemical Staining

Human prostate tissue samples from the paraffin embedded whole mount blocks from the 43 patients originally characterized for BPH pathology severity and utilized for corresponding TZ derived RNA were obtained from the Vanderbilt Tissue Acquisition Core via the Department of Pathology and tissue microarrays were generated by one of the authors (MPR). The microarrays contained three 0.6 mm core samples, two from the TZ and one from PZ away from the cancer involved area. Staining of tissue sections was performed using a previously described protocol [29]. Sections (5 µm) were cut and mounted on charged glass slides. After deparaffinization and rehydration, the tissue sections were subjected to antigen retrieval by heating in a microwave for 10 minutes in Vector H-3300 antigen unmasking solution (1∶100; Vector Laboratories, Inc., Burlingame, CA). Slides were then incubated in 0.3% hydrogen peroxide in methanol for 30 minutes at room temperature (RT), followed by a 1 hr incubation in 5% goat serum in PBS at RT. Slides were incubated with primary antibodies overnight at 4°C in 5% goat serum in PBS. Antibodies used were a mouse monoclonal against TIMP2 (1∶300, sc-21735, Santa Cruz Biotechnology, Santa Cruz, CA), and rabbit polyclonals against FGF2 (1∶500, sc-79, Santa Cruz Biotechnology, Santa Cruz, CA), and SMOC1 (1∶100, Atlas Antibodies, Stockholm, Sweden). The slides were then washed and incubated in a biotinylated secondary antibody (rabbit anti-mouse or swine anti-rabbit, 1∶300, DAKO, Carpinteria, CA) at RT for 60 min, washed in PBS extensively, then incubated in ABC-HRP complex (Vector Laboratories) for 30 min. Bound antibodies were then visualized by incubation with liquid 3,3′-diaminobenzidine tetrahydrochloride (DAKO). Slides were then rinsed extensively in tap water, counterstained with hematoxylin, and mounted. Stained tissue sections were photographed and processed using a Zeiss AX10 Imager.M1 microscope and AxioVision Release 4.6 software.

The immunostained slides were reviewed by a pathologist (MPR) blinded to the BPH pathology severity of the case and results expressed in a semi-quantitative manner. Percentage staining was scored on a scale of 0 to 4, where 0 = no staining, 1 = less than 25%, 2 = 25% to 50%, 3 = 50% to 75%, and 4 = 75% to 100%. The intensity of staining was scored on a scale of 1 to 3, where 1 = mild, 2 = moderate, and 3 = marked. For a sample yielding no staining, the intensity score was also 0. When both stromal and epithelial staining was present, scoring was done separately. To calculate a protein expression index, the percentage score was summed with the intensity score. The combined scores were plotted for each BPH pathology category (minimal, mild, moderate, severe BPH changes), and Kruskal-Wallis tests were used to compare the combined scores across different disease severity status.

## Results

### Androgen-Regulated Gene Expression Profiles in Human Prostate Transition Zone

**Figure 1:**
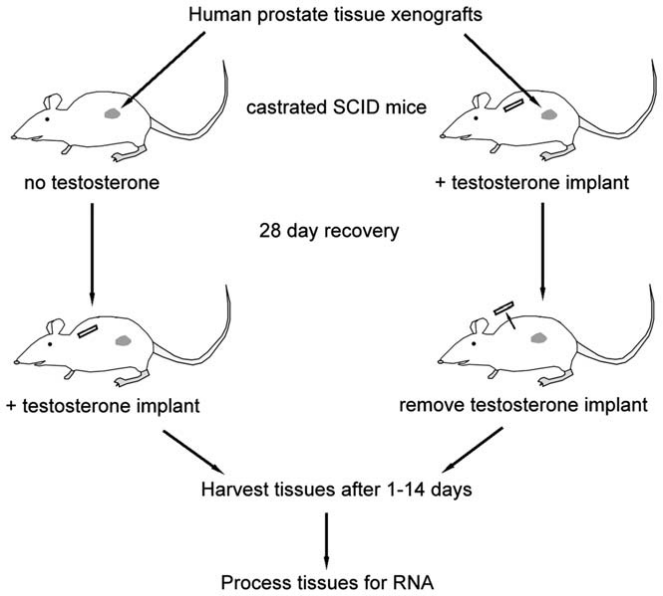
Experimental scheme for addition or withdrawal of testosterone in human prostate xenografts. TZ tissues from six patients were xenografted beneath the renal capsules of castrated male SCID mice (10 mice per patient). Five mice from each group were then given sub-cutaneous implants containing 25 mg of testosterone. After allowing the xenographs to establish for one month, the implants were removed from the testosterone supplemented mice, and 25 mg testosterone pellets were implanted in mice that had not received testosterone. Control mice were sacrificed at the time of androgen addition or removal, and the remaining mice from each group were sacrificed at 1, 3, 7, and 14 days.

We profiled gene expression patterns from human prostate TZ xenografts, following the addition or subtraction of testosterone. Figure 1 illustrates the general scheme used for manipulation of androgen levels in SCID mice harboring sub-renal capsule prostate xenografts. Total RNA was prepared from harvested xenografts with or without exposure to testosterone for 0, 1, 3, 7, and 14 days. A pool made from equal amounts of RNA from day 0 castrate and intact mouse xenografts was used as a standard reference. A total of 58 hybridizations were completed, which included samples from six patients. Initial analysis indicated a high level (up to 10 fold) of patient-to-patient variation in expression levels in known androgen regulated genes such as *KLK3* (PSA). In order to allow for this expected biologic variability between individual patient tissues, each patient's tissue derived RNA samples were re-centered to that patient's time 0 controls. For the primary analysis, two time points were considered, days 0 and 14, yielding 5,679 genes with a 1.5 fold or greater increase in expression. An ANOVA analysis was performed on these 5,679 genes, with a *P*-value cut-off of 0.05, which predicts 284 false positives. Genes that had at least a 1.5-fold increase or decrease in expression in the day 14 sample vs. day 0 were then filtered by signal expression value, retaining the top 95% of signal dynamic range, yielding 784 genes. The microarray data have been deposited in NCBI's Gene Expression Omnibus [30] and are accessible through GEO Series accession number GSE17862 (http://www.ncbi.nlm.nih.gov/geo/query/acc.cgi?acc=GSE17862).

### Genes Up-Regulated by Androgens in Human TZ Xenografts

Table 1 lists the top 45 genes that were up-regulated in response to androgen, sorted by fold change (in descending order). The top three genes, *KLK3* (PSA), *MSMB* (beta-microseminoprotein), and *TMEPAI* (transmembrane prostate androgen-induced protein) were all previously well known to be regulated by androgens in human prostate [31], [32], [33]. Several other genes identified as up-regulated in human TZ xenografts by testosterone have also been previously shown to be regulated by androgens. These include *TRPM8* (transient receptor potential melastatin member 8), a putative prognostic marker and therapeutic target in prostate cancer [34], [35], *ACPP* (prostatic acid phosphatase) [36], *SCGB1A1* (uteroglobin) [37], *NDRG1* (N-myc downstream regulated gene 1) [38], and *CREB3L4* (cAMP responsive element binding protein 3-like 4) [39]. *RNASEL* has been reported as a candidate hereditary prostate cancer gene [40]. Other genes identified that have been reported as androgen-regulated or involved in BPH or prostate cancer included *PTGDS* (prostaglandin D2 synthase), *FGFBP1* (fibroblast growth factor binding protein 1), *KLK11* (kallikrein 11), *CXCL5* (chemokine (C-X-C motif) ligand 5), *FKBP11* (FK506-binding protein 11) and *TIMP1* (tissue inhibitor of metalloproteinase 1).

**Table 1:** Genes induced in response to androgen supplementation, day 0 vs. day 14.

Accession no.	Fold Change	Symbol	*P*-value	Gene Name
NM_001648	14.27	KLK3	0.00048	prostate specific antigen
NM_002443	5.31	MSMB	0.0488	beta-microseminoprotein
NM_020182	5.09	TMEPAI	>0.05	transmembrane prostate androgen-induced protein
AL049919	3.59	CUTL2	0.00761	cut-like homeobox 2
AY090780	3.35	LOC131368	>0.05	hypothetical protein LOC131368
NM_007281	3.25	SCRG1	0.0115	scrapie responsive protein 1
NM_144597	3.23	MGC29937	0.00971	hypothetical protein MGC29937
AF469196	3.22	NUDT10	0.0478	nudix-type motif 10
NM_024080	3.19	TRPM8	0.00903	transient receptor potential melastatin member 8
NM_014405	3.16	CACNG4	0.000776	voltage-dependent calcium channel gamma-4 subunit
NM_014244	3.15	ADAMTS2	0.00006	ADAM with thrombospondin motifs-2
NM_170601	3.12	CSE-C	0.00813	cytosolic sialic acid 9-O-acetylesterase homolog
NM_002212	3.06	EIF6	>0.05	Eucaryotic initiation factor 6
NM_003504	3.03	CDC45L	0.00298	CDC45-like
D17189	2.98	GOLGA3	0.00791	golgi autoantigen, golgin subfamily a, 3
NM_001099	2.96	ACPP	0.0168	prostatic acid phosphatase
AF086356	2.89	TMEM59	>0.05	transmembrane protein 59
NM_003104	2.87	SORD	0.00453	sorbitol dehydrogenase
NM_003357	2.86	SCGB1A1	0.000497	secretoglobin, family 1A, member 1 (uteroglobin)
NM_001082	2.85	CYP4F2	0.000759	cytochrome P450, family 4, subfamily F, polypeptide 2
NM_022137	2.76	SMOC1	0.00747	secreted modular calcium-binding protein 1
NM_006877	2.76	GMPR	0.000878	guanosine monophosphate reductase
NM_000180	2.73	GUCY2D	0.0136	guanylate cyclase 2D, membrane (retina-specific)
NM_002465	2.73	MYBPC1	0.0126	myosin binding protein C, slow type isoform 1
BC032412	2.72	MGC40574	0.021	MGC40574 protein
NM_022471	2.69	GMCL1L	>0.05	germ cell-less homolog 1 (Drosophila)-like
NM_152284	2.68	SHAX3	0.000453	Snf7 homologue associated with Alix 3
NM_152590	2.67	FLJ36004	0.0232	hypothetical protein FLJ36004
NM_152351	2.66	SLC5A10	>0.05	solute carrier family 5, member 10
NM_014353	2.61	RAB26	>0.05	RAB26, member RAS oncogene family
NM_006096	2.59	NDRG1	0.0415	N-myc downstream regulated gene 1
AK024944	2.56	MED28	0.00285	mediator complex subunit 28
AF118078	2.55	PRO1848	0.00611	PRO1848
AB051448	2.53	KIAA1661	>0.05	KIAA1661 protein
U92012	2.52	PDS5A	>0.05	PDS5, regulator of cohesion maintenance, homolog A
BC039353	2.49	TTLL1	>0.05	tubulin tyrosine ligase-like family, member 1
NM_052863	2.45	SCGB3A1	0.00228	secretoglobin, family 3A, member 1
NM_024980	2.44	GPR157	>0.05	G protein-coupled receptor 157
NM_130898	2.41	CREB3L4	0.00235	cAMP responsive element binding protein 3-like 4
NM_006820	2.41	IFI44L	0.0139	interferon-induced protein 44-like
NM_003027	2.40	SH3GL3	>0.05	SH3-domain GRB2-like 3
NM_021133	2.38	RNASEL	>0.05	ribonuclease L

*TMEPAI* (transmembrane prostate androgen-induced protein) was the most highly expressed androgen-regulated gene identified. Additional highly expressed, androgen-regulated genes included *KLK3* (PSA), *MSMB* (beta-microseminoprotein), *ACPP* (prostatic acid phosphatase) and *SCGB1A1* (uteroglobin).

### Genes Up-Regulated in Response to Androgen Withdrawal

Table 2 lists the top 45 genes that were up-regulated in response to the withdrawal of androgen for 14 days, sorted by fold change (in descending order). One of the genes identified, *GLI1* is up-regulated in mouse prostate following castration [41]. Another gene identified with increased expression was *ANNAT1* (annexin 1), which has been reported to decrease in androgen stimulated prostate cancer compared with benign prostatic epithelium [42].

**Table 2:** Genes induced in response to androgen withdrawal, day 0 vs. day 14.

Accession no.	Fold Change	Symbol	*P*-value	Gene Name
L19363	5.09		0.00151	radioresistant malignant melanoma cDNA
NM_015161	4.20	ARL6IP	0.0345	ADP-ribosylation factor-like 6 interacting protein
NM_007018	4.17	CEP1	0.024	centrosomal protein 1
NM_033337	4.17	CAV3	0.00936	caveolin 3
AK090628	4.09		0.00654	Neuroglioma cDNA
NM_000259	4.05	MYO5A	0.0195	myosin VA (heavy polypeptide 12, myoxin)
AK058128	3.49		0.0148	testis cDNA
NM_000700	3.48	ANXA1	0.00759	annexin I
NM_013278	3.35	IL17C	0.0164	interleukin 17C
BC015390	3.31		0.0205	unknown (protein for IMAGE:4403366)
NM_003758	3.09	EIF3S1	0.0369	eukaryotic translation initiation factor 3, subunit 1 alpha
NM_005042	3.07	PRH2	0.0317	proline-rich protein HaeIII subfamily 2
NM_020191	3.01	MRPS22	0.0288	mitochondrial ribosomal protein S22
NM_020403	2.97	PCDH9	0.0394	protocadherin 9 isoform 2
AY090737	2.96	CNTN4	0.00783	contactin 4
BQ025872	2.94	MYO9A	0.0267	myosin IXA
NM_005269	2.88	GLI 1	0.0202	glioma-associated oncogene homolog 1
NM_001938	2.86	DR1	0.0197	down-regulator of transcription 1
NM_080387	2.85	CLECSF8	0.0456	C-type lectin, superfamily member 8
AL833691	2.83	AFF3	0.0195	AF4/FMR2 family, member 3
NM_018365	2.83	MNS1	0.042	meiosis-specific nuclear structural protein 1
AK056076	2.81	FLJ31514	0.00423	hypothetical protein
AL832652	2.79	DNAH17	0.0269	dynein, axonemal, heavy chain 17
NM_152390	2.79	TMEM178	0.0329	transmembrane protein 178
BF111085	2.79	SOX1	0.0387	SRY (sex determining region Y)-box 1
NM_012311	2.78	KIN	0.047	HsKin17 protein
AF086063	2.77	BCL2	0.0119	B-cell CLL/lymphoma 2
NM_080629	2.74	COL11A1	0.0364	alpha 1 type XI collagen isoform A
AF324830	2.73	LIR9	0.0377	leukocyte Ig-like receptor 9
AB033091	2.71	SLC39A10	0.0495	solute carrier family 39 (zinc), member 10
BC033949	2.68		0.022	
NM_003704	2.62	C4orf8	0.028	res4-22 protein
NM_004934	2.61	CDH18	0.0161	cadherin 18, type 2
NM_014221	2.60	MTCP1	0.0367	mature T-cell proliferation 1
NM_025170	2.57	DEPDC2	0.0114	DEP domain containing 2
NM_007072	2.56	HHLA2	0.0262	HERV-H LTR-associating 2
NM_014574	2.52	STRN3	0.0267	striatin, calmodulin binding protein 3
NM_015102	2.52	NPHP4	0.0194	nephronophthisis 4
NM_013943	2.51	CLIC4	0.0483	chloride intracellular channel 4
NM_017653	2.40	DYM	0.00184	dymeclin
NM_147175	2.39	HS6ST2	0.0196	heparan sulfate 6-O-sulfotransferase 2
NM_025140	2.33	CCDC92	0.0368	coiled-coil domain containing 92
NM_052946	2.31	NOSTRIN	0.00289	nitric oxide synthase trafficker
NM_005108	2.29	XYLB	0.00043	xylulokinase homolog
NM_025208	2.27	PDGFD	0.0444	platelet derived growth factor D

### Quantitative RT-PCR Analysis of Human Prostate RNA Pools

To further investigate the expression levels of androgen regulated genes identified by microarray analysis, quantitative RT-PCR analysis was performed on selected targets using RNA pools derived from human prostate tissues. A major clinical need is to identify biomarkers that provide prognostic information regarding BPH progression or that may predict response to treatments, including 5 alpha reductase inhibitors. Secreted proteins that can be readily measured in blood are particularly attractive clinical targets. From the gene expression profiles in hormone manipulated TZ xenografts, potential biomarkers were systematically selected with overlapping bioinformatics criteria, employing WebGestalt. Gene targets selected were 1) androgen-regulated within our microarray data, 2) expressed at significantly higher levels in the prostate relative to other tissues, and 3) known or predicted to express secreted or cell surface proteins. These criteria yielded a set of candidate genes (Table 3) that included some previously known biomarkers, including *KLK3*, *ACPP*, and *MSMB*, further validating our experimental approach. Other genes of interest added “manually” for RT-PCR analysis were *IGF1*, *IGF1R*, *TGFB1*, *TGFB3*, *TGFBR1*, and *TGFBR2*. The expression levels of these target genes were measured in RNA pools prepared from TZ tissues designated as having mild, moderate, and severe BPH changes, as well as moderately and poorly differentiated prostate cancer. RNA for each category of BPH or prostate cancer tissues was pooled from 5–10 patient samples. Control RNA was pooled from TZ samples from patients with no appreciable TZ expansion from glandular and stromal hyperplasia.

**Table 3:** Selection criteria for candidate biomarker targets.

Symbol	Fold Increase T14	*P* Increase T14	Fold Decrease C14	*P* Decrease C14	Cell surface?	Over Expr in Prostate?
CART	1.91				Y	Y
FGFBP1	2.05	0.0165			Y	Y
SGCD	1.70				Y	Y
EMP3	1.99	0.0171			Y	Y
KLK11	1.92	0.0392			Y	Y
ACPP	2.96	0.0168			Y	Y
ALCAM	1.65				Y	Y
ANK1		0.0219			Y	Y
CHRNB1		0.0292			Y	Y
CNFN	1.67	0.00136			Y	Y
CNTNAP2	2.01	0.0151			Y	Y
EFEMP2	1.69	0.00255			Y	Y
F10	1.98	0.00321			Y	Y
F11R			0.59		Y	Y
FBN1		0.0223			Y	Y
GABRG2	1.56				Y	Y
HS3ST4	1.52				Y	Y
IGF2					Y	Y
IGF2R		0.017			Y	Y
IHH	1.55				Y	Y
KLK3	14.27	0.00048			Y	Y
LIPG	1.76				Y	Y
MGP			0.59		Y	Y
MSMB	5.31	0.0488			Y	Y
SCGB1A1	2.86	0.000497			Y	Y
SCGB3A1	2.45	0.00228			Y	Y
SEMA4F	2.03				Y	Y
SGCA	1.53	0.0161			Y	Y
SLC26A2	1.53				Y	Y
SMOC1	2.76	0.00747	0.57		Y	Y
STEAP	1.58				Y	Y
TMEPAI	5.09				Y	Y
WNT5B	1.56	0.00478			Y	Y
HIST1H2AE	1.55	0.0138				Y
MAP2K5	1.64	0.0297				Y
RPS6KA2	1.53					Y
HIPK2	1.58	0.0372				Y
RBM42		0.000721				Y
AP1B1	1.54	0.00222				Y
C9orf61	1.97	0.0115				Y
COMT	1.59	0.0295				Y
COX5B		0.00132				Y
CPNE4	2.18	0.033	0.42			Y
CTTN	1.62	0.00328				Y
CYP1B1	1.94	0.0452	0.32	0.0289		Y
DAP13		0.0417				Y
PAAF1			0.63			Y
FLJ22795	2.08					Y
GABARAP		0.0358				Y
MAOA	1.88	0.0124				Y
MARCKS			0.65			Y
FRMD5	1.63	0.00218				Y
NDUFA2		0.0358				Y
NME2		0.00538	0.65			Y
NMES1	1.94					Y
PSMB4		0.00151				Y
RNASEL	2.38		0.10	0.0412		Y
RPL30			0.64			Y
RPLP2		0.0412				Y
RPS26			0.55			Y
S100A11		0.0204				Y
S100A6		0.0443				Y
SEC24B			0.63			Y
SELM	1.57	0.0347				Y
SSR4	1.94	0.000199				Y
TXN2		0.0221				Y
TXNDC9	1.89					Y
VKORC1	1.55					Y
CDH13			0.52		Y	
ALG10	1.57				Y	
COL4A5	1.63				Y	
FGF2			0.47		Y	
RTN3			0.62		Y	
TGFB2	1.54				Y	
TIMP2			0.55		Y	
RARB			0.62			
UGCG			0.66		N, Y?	
FUZ	2.01				N, Y?	
IGF1					Y	
IGF1R					Y	
TGFB1					Y	
TGFB3					Y	
TGFBR1					Y	
TGFBR2					Y	
18S rRNA						

Potential candidates from our microarray results (androgen-regulated ≥1.5-fold or *P*≤0.05) were combined with systematically selected genes using overlapping bioinformatics criteria, employing WebGestalt. T14 = testosterone, day 14 and C14 = castrate, day 14.

The results of the qRT-PCR analyses are shown in Table 4. RNA expression levels were determined for 86 candidate biomarker genes. Of these, 72 genes were determined to have a greater than 6-fold higher level than the normal control pool in at least one of the BPH or prostate cancer pools. Relative to the control prostate RNA pool, the average levels in the BPH or prostate cancer RNA pools with expression levels <1-fold were designated as “low”, 1- to 6-fold were designated as “moderate”, and >6-fold were designated as “high”. Based on these criteria, each gene was assigned to one of six categories (Table 4). Fifteen genes were high in both BPH and prostate cancer, 41 genes were high in BPH and moderate in prostate cancer, two genes were high in BPH and low in prostate cancer, and five genes were moderate in BPH and high in prostate cancer. No genes were found that were classifiable as “low” in BPH.

**Table 4:** Real-time qRT-PCR analysis in RNA pools derived from BPH and prostate cancer.

Category	Gene	Mild	Mod	Sev	CaP	BPH/CaP
**Low in Prostate Cancer**						
Mod in BPH	SCGB1A1	4.7	0.4	1.4	0.1	15.5
	SCGB3A1	2.4	0.4	0.5	0.1	13.8
	MGC14161	1.5	0.7	1.0	0.5	2.3
High in BPH	SMOC1	12.0	10.7	17.7	0.5	25.9
	S100A6	9.1	6.7	8.0	0.6	13.2
**Moderate in Prostate Cancer**						
Mod in BPH	TGFBR1	4.8	5.1	6.1	2.5	2.1
	F11R	6.8	3.3	7.2	3.4	1.7
	DAP13	2.7	3.9	4.9	2.8	1.4
	PSMB4	2.1	1.5	2.5	1.5	1.4
	RTN3	6.0	5.6	6.1	4.5	1.3
	VKORC1	5.4	5.0	4.8	3.8	1.3
	ALG10	4.6	3.6	4.6	4.4	1.0
	IHH	5.6	1.4	6.5	5.3	0.9
	RPS26	4.0	3.0	2.5	3.6	0.9
	SLC26A2	3.6	2.4	4.7	5.4	0.7
	ALCAM	4.0	2.5	3.1	5.3	0.6
	CART	0.7	0.3	1.5	2.3	0.4
	CYP1B1	2.5	1.4	2.0	4.6	0.4
High in BPH	IGF2	32.9	16.3	39.8	1.6	18.1
	F10	21.1	11.0	18.5	1.0	16.6
	TGFB3	12.3	15.3	18.6	1.1	14.7
	FGFBP1	24.6	1.6	10.6	1.2	10.2
	FGF2	8.5	9.9	11.3	1.1	9.2
	EMP3	9.8	7.3	10.1	1.0	8.7
	SGCA	4.5	12.6	7.4	1.1	7.8
	IGF1	20.2	20.7	31.7	3.7	6.6
	TGFBR2	14.2	9.0	11.4	2.0	5.8
	LIPG	24.2	10.4	40.7	4.6	5.4
	TIMP2	9.6	11.6	11.9	2.1	5.3
	RARB	14.0	7.2	11.4	2.2	5.0
	RARB	7.0	4.9	5.4	1.3	4.5
	EFEMP2	8.6	7.5	6.3	1.6	4.5
	SGCD	4.8	7.9	9.0	2.0	3.7
	RNASEL	9.2	6.3	9.7	2.3	3.6
	SELM	6.9	6.9	7.3	2.0	3.6
	COL4A5	9.1	5.9	10.7	2.6	3.3
	TGFB1	12.1	9.0	8.8	3.3	3.1
	CDH13	10.5	7.1	10.3	3.1	3.0
	CHRNB1	9.1	6.7	10.5	2.9	3.0
	ACPP	6.5	5.6	16.6	3.2	2.9
	TGFB2	4.1	6.7	7.3	2.2	2.8
	MGP	6.5	13.8	11.8	4.2	2.6
	SEMA4F	12.6	9.1	15.1	4.8	2.5
	UGCG	8.8	7.6	9.0	4.1	2.4
	TGFB2	4.2	6.4	5.2	2.3	2.3
	WNT5B	12.0	7.2	10.9	4.6	2.2
	COMT	11.4	7.3	13.7	5.3	2.1
	MSMB	2.7	4.1	11.7	3.0	2.1
	SEC24B	8.3	6.1	9.9	4.1	2.0
	AP1B1	8.3	6.5	14.0	5.0	1.9
	MARCKS	9.5	9.4	14.5	5.9	1.9
	MSMB	1.7	2.6	8.6	2.3	1.9
	COX5B	7.6	6.9	12.1	4.9	1.8
	NDUFA2	6.9	5.5	13.1	4.6	1.8
	FLJ11848	6.1	5.5	6.6	3.6	1.7
	RPS6KA2	7.7	7.1	12.0	5.4	1.7
	IGF2R	6.3	5.4	11.4	4.8	1.6
	MAP2K5	8.2	8.6	8.3	5.1	1.6
	CTTN	6.9	4.8	8.8	4.5	1.5
	GABARAP	7.6	5.7	7.4	5.2	1.3
	S100A11	8.0	4.3	7.6	5.1	1.3
	IGF1R	5.4	3.5	9.8	5.3	1.2
**High in Prostate Cancer**						
Mod in BPH	NME1	6.9	4.4	6.6	7.8	0.8
	CPNE4	3.3	3.5	8.5	7.2	0.7
	STEAP	4.2	2.9	6.5	6.6	0.7
	C9orf61	4.3	3.9	6.6	9.0	0.5
	NMES1	4.8	1.2	4.4	18.1	0.2
High in BPH	MGC10433	13.5	11.7	16.6	6.6	2.1
	TXN2	9.8	7.9	11.7	6.6	1.5
	FLJ22688	14.4	6.9	10.4	7.8	1.4
	RPL30	13.3	9.6	14.1	10.5	1.2
	SSR4	7.1	6.0	10.7	6.4	1.2
	MAOA	9.6	4.3	9.4	6.9	1.1
	TMEPAI	7.5	8.6	17.7	10.0	1.1
	HIST1H2AE	6.5	5.6	8.7	6.7	1.0
	RPLP2	8.1	5.6	7.4	6.9	1.0
	HIPK2	8.4	7.5	11.5	10.4	0.9
	KLK3	3.3	4.9	13.3	7.7	0.9
	CNTNAP2	4.2	10.9	12.6	12.7	0.7
	FLJ22795	12.0	6.2	9.8	13.1	0.7
	HS3ST4	7.1	4.0	12.2	10.6	0.7
	KLK11	14.2	8.6	9.1	17.0	0.6
	KLK11	9.9	4.6	5.2	16.9	0.4
	KLK3	7.0	7.1	22.5	30.7	0.4

Comparison between gene status in BPH and prostate cancer. BPH categories are mild, moderate (Mod), and severe (Sev) and prostate cancer (CaP) pools are derived from both moderately and poorly differentiated tissues. Average expression levels <1-fold were defined as “low”, levels >6-fold were defined as “high”, and levels between 1-fold and 6-fold were defined as “moderate”. Expression levels are relative to a control pool derived from TZ samples from patients with no appreciable TZ expansion. Genes that appear twice were measured using two distinct primer sets. BPH/CaP is the ratio of the average levels in the BPH pools divided by the average levels in the prostate cancer pools.

### Candidate BPH Biomarker Genes

A number of genes had expression levels in the BPH pools that increased with severity of disease. Some of the genes had expression levels that were particularly elevated in BPH, but relatively lower in the prostate cancer pools. These genes would be expected to have the best potential as candidate BPH biomarkers, including specificity compared to prostate carcinoma. Those expressed extracellularly and with an average expression level at least three-fold greater in BPH RNA pools than in the prostate cancer RNA pools included: *CDH13*, *CHRNB1*, *COL4A5*, *EFEMP2*, *EMP3*, *F10* (16-fold), *FGF2*, *FGFBP1* (10-fold), *IGF1*, *IGF2* (18-fold), *LIPG*, *RARB*, *SGCA*, *SGCD*, *TGFB1*, *TGFB3* (14-fold), *TGFBR2* and *TIMP2* of the “high in BPH, moderate in prostate cancer” group; *SMOC1* (26-fold) in the “high in BPH, low in prostate cancer” group; and *SCGB3A1* (15-fold) and *SCGB1A1* (14-fold) in the “moderate in BPH, low in prostate cancer” group.

### Immunohistochemical Staining for Select Gene Targets in Pathology-Characterized BPH Tissues

**Figure 2:**
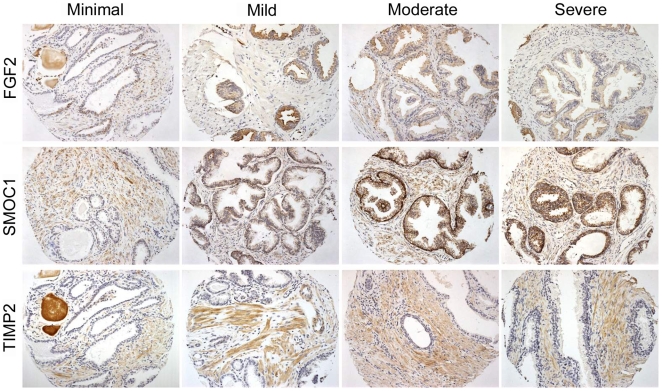
Immunohistochemical staining for FGF2, SMOC1, and TIMP2 in BPH samples with different degrees of severity. Tissue sections are ∼0.6 mm in diameter. FGF2 and SMOC1 staining was observed in both stromal and epithelial cells while TIMP2 staining was present predominantly in stromal cells.

To determine if the differences in the RNA expression levels observed in BPH tissues were also present at the protein level and to further characterize the epithelial and/or stromal localization of potential increased expression, we performed immunohistochemistry (IHC) on a BPH tissue microarray (TMA). The array contained 129 tissue sections from 43 unique patients, containing BPH tissues with pathologies ranging from minimal to severe as defined in Materials and Methods. *FGF2*, *SMOC1* and *TIMP2* were chosen for IHC testing, as these genes had markedly increased RNA expression levels in BPH relative to controls, and antibodies were available that produced specific and satisfactory staining of human TZ tissues. Representative immunostaining for each antibody is shown in Figure 2. TIMP2 immunostaining was observed predominantly in stromal cells, while FGF2 and SMOC1 staining was observed in both stromal and epithelial cells. The immunostaining for TIMP2, FGF2, and SMOC1 was often quite marked. Additionally, we immunostained some larger conventional histology sections of BPH tissue using all three antibodies, and observed that the staining was frequently focal and variable (data not shown), compatible with observations made with TMA slides.

**Figure 3:**
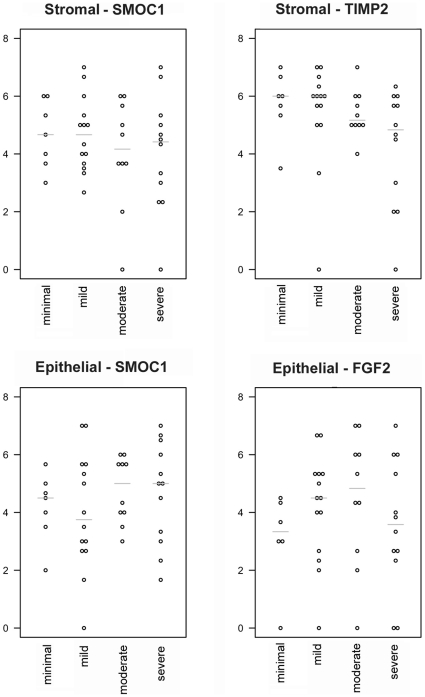
Immunohistochemical staining by disease severity category. Percentage staining was scored on a scale of 0 to 4, where 0 = no staining, 1 = less than 25%, 2 = 25% to 50%, 3 = 50% to 75%, and 4 = 75% to 100%. The intensity of staining was scored on a scale of 1 to 3, where 1 = mild, 2 = moderate, and 3 = marked. For a sample yielding no staining, the intensity score was 0. When both stromal and epithelial staining was present, scoring was done separately. For the samples for which two measurements were available, the average score was used to represent the sample. To calculate a protein expression index, the percentage score was summed with the intensity score; the maximum score was 7. The horizontal gray line represents within-group median.

To more objectively analyze immunostaining results and allow for statistical analysis, immunoreactivity on TMA sections was scored semiquantitatively as described in the Materials and Methods section. On the scale of 0–7 for combined extent and intensity, the majority of samples that had duplicate TMA core sections to assess had scores for individual sections separated by at most 1 as shown in Table 5, and the average score for duplicate sections was used for each case for statistical purposes. As shown in Figure 3, none of the three examined proteins were found to be significantly associated with BPH pathology severity based on this limited IHC analysis. Kruskal-Wallis tests for overall difference yield *P*-values ranging from 0.10 to 0.96.

**Table 5:** Percentages of immunostaining scores from duplicated samples separated by more than 1.

	Protein	Percentage Score	Intensity Score
Stromal	smoc1	3/43 (7.0)	1/41 (2.4)
	calcyclin	4/43 (9.3)	1/17 (5.9)
	timp2	3/43 (7.0)	0/41 (0.0)
Epithelial	smoc1	7/43 (16.3)	0/42 (0.0)
	calcyclin	0/43 (0.0)	0/6 (0.0)
	fgf2	7/43 (16.3)	0/36 (0.0)

Tissue sections were scored on a scale of 0–7 for combined extent and intensity. The ratios listed are the number of duplicate sections with scores differing by more than 1, divided by the total number of sections scored. Intensity is scored only on the samples for which percentage score is greater than 0.

## Discussion

There is a need for BPH-specific markers that correlate with disease progression, which could be used for the early identification of patients likely to progress to more severely symptomatic disease. The timely use of existing or novel non-surgical therapies in these patients may reduce the significant cost and morbidity of surgical intervention. Hyperplastic growth of the prostate transition zone associated with clinical BPH may be the result of the abnormal expression of key androgen responsive genes, including those involved in prostatic development, and which lead to an imbalance between cell division and cell death. Although several candidate mediators have been suggested to play a role in BPH, the androgen regulated genes that are important for both normal and abnormal prostate growth remain to be completely defined. We have identified a panel of genes regulated by androgens in human transition zone prostate tissue *in vivo*. The expression of a subset of these genes was correlated at the RNA level with disease status in BPH tissues.

A subset of these genes was investigated at the protein level by IHC. Expression in epithelium and/or stroma was confirmed in tissues of varying severity BPH pathology. However, no obvious correlation was seen between IHC intensity and BPH pathology severity. This panel of genes provides a valuable dataset for androgen regulated genes that could be etiologically involved in the abnormal epithelial and stromal proliferation characteristic of BPH. Future investigation will be necessary to establish the biologic significance of specific genes in the evolution of BPH and the possible suitability as a target for pharmacologic intervention. It is desirable to have biomarkers of clinical utility in BPH prior to acquisition of tissue as a consequence of therapeutic intervention (e.g., TURP or suprapubic prostatectomy). As such, tissue based quantitation of mRNA levels or IHC intensity is unlikely to be a clinically useful test. Our studies provide a candidate list for further investigation using more suitable test substrates, such as urine for mRNA or protein or serum for protein. The highly relevant model of androgen regulated expression in intact TZ tissues analogous to actual BPH tissues increases the likelihood that such candidate targets could have prognostic or therapeutic relevance.

We used oligonucleotide microarrays to examine the expression of androgen regulated genes in normal human TZ prostate tissue growing as xenografts in male SCID mice. Among genes with androgen regulated expression that we identified, those with higher expression in prostate relative to other tissues and those of the cell surface or extracellular compartment have greatest potential to serve as useful clinical biomarkers. The most promising candidates were culled with WebGestalt bioinformatics tools. The expression of these genes was screened using qRT-PCR analysis of cDNA pools derived from BPH or prostate cancer tissues, to determine whether expression was correlated with disease status.

When generating the androgen regulated gene lists, we used a 14 day time point in order to look for genes chronically up-regulated in response to androgens. Genes identified as up-regulated by androgens at this longest time point investigated were also generally found to gradually increase levels at earlier time points. Genes transiently up-regulated at earlier time points may reflect genes indirectly induced during growth and differentiation, and would likely be poor choices for markers of prostatic disease.

Tissue obtained as a surgical specimen from prostatectomy can be subjected to prolonged blood supply interruption during the surgical procedure, compromising RNA quality. The use of xenografts allows the tissue to recover from damage induced during surgery. Xenografts can be harvested rapidly with less severe ischemia, thus allowing for the isolation of high quality RNA. In addition to producing high quality tissue, this method vs use of cell lines also allows us to study tissues with intact stromal-epithelial interactions occurring *in vivo*. However, since the exact ratios of stroma to epithelia are variable, genes identified as altered at the RNA level in intact tissues could be related to changes in epithelium, stroma or both. A weakness of the method is that the tissue used for RNA extraction is destroyed, and even adjacent tissues from the same patient may have markedly differing stromal-epithelial ratios. Therefore, this approach requires subsequent validation by *in situ* hybridization or immunohistochemistry to determine cell type specificity.

Several genes identified in our microarray analysis were previously known to be regulated by androgens or involved in prostatic disease. These included the up-regulated genes *ACPP*, *CXCL5*, *FGFBP1*, *FKBP11*, *KLK11*, *PTGDS*, and *TIMP1*. CXCL5 was recently shown to be elevated in serum from patients with BPH and may potentially distinguish between BPH and prostate cancer among patients presenting with low serum PSA [43]. However, CXCL5 was also recently shown to promote prostate cancer progression [44]. FGFBP1 is secreted from AR+ PC3 cells in response to androgen [45], and is highly expressed in some human prostate tumor cells and the proliferation of these cells was dependent on these high expression levels [46]. *FKBP11* expression was up-regulated in mouse prostate by androgen [47]. Numerous studies have investigated the use of serum levels of KLK11 as a diagnostic marker to discriminate between prostate cancer and BPH [48], [49], [50]. PTGDS derived prostaglandin D2 produced by prostate stromal cells suppresses the growth of prostate tumor cells through a PPARgamma-dependent mechanism [51]. TIMP1 levels were significantly elevated in blood plasma from prostate cancer patients with metastases [52]. Such genes shown to be androgen regulated and possibly showing altered protein levels in sera of BPH patients are of interest as possible biomarkers in BPH. However, substantial additional work is necessary to investigate the possible role of these genes in the etiology of altered epithelial and stromal proliferation in the TZ of BPH patients, their possible contribution to the symptomatology of clinical BPH, and their suitability as candidate biomarkers in the management of BPH.

We also identified genes in our microarray analysis that were down-regulated by androgens. These included *GLI1*, *ANNAT1* (annexin 1), *BCL2*, and *CLIC4*. Compatible with our observations, expression of *GLI1* was reported to increase in mouse prostate after the withdrawal of androgen [41]. The expression of annexin 1 has been reported to decrease in androgen stimulated prostate cancer compared with benign prostatic epithelium [42]. Annexin 1 was also reported to be highly over expressed in androgen-independent LNCaP cells compared to androgen-dependent LNCaP cells [53]. *BCL2* and *CLIC4*, have been reported to have anti-apoptotic, and proapoptotic activities, respectively. Cardillo et al. found elevated *BCL2* expression in apoptosis resistant BPH following androgen deprivation [54]. *CLIC4* is involved in p53 mediated apoptosis [55]. At 14 days post androgen withdrawal the acute apoptotic phase of tissue rearrangement in prostate xenografts has been largely completed [22]. However, genes with decreased expression in androgen stimulated TZ xenografts could be relevant in the pathophysiology of androgen related abnormal glandular and stromal growth in BPH and may be important to investigate further.

Serum PSA commonly used for prostate cancer screening is clearly related to prostate volume, which is increased as a consequence of TZ expansion due to glandular and stromal hyperplasia seen in BPH. Biomarkers of greater specificity for either CaP or BPH are greatly needed. Fourteen genes were identified as being highly elevated in BPH and had BPH/PCa expression ratios of at least 5-fold (table 4). Of these genes, eight have been previously implicated in BPH or prostate cancer, and are also expressed extracellularly. These are *EMP3*, *FGF2*, *FGFBP1*, *IGF1*, *IGF2*, *TGFB3*, *TGFBR2*, and *TIMP2*. *FGF2* (fibroblast growth factor 2), *IGF1* (insulin-like growth factor 1), and *IGF2* (insulin-like growth factor 2), have altered expression levels in BPH and prostate cancer [56], [57] and men with elevated IGF-1 serum levels have an increased risk for BPH [58]. IGF-II serum levels increase the discrimination between BPH and prostate cancer and improve the predictive value of PSA in clinical staging [59]. TGFβ3 (transforming growth-factor β3) is expressed in BPH and normal prostate basal epithelial cells, but is reduced or absent in prostate cancer [60].

Other genes identified from the microarray analysis as androgen regulated and that were also highly expressed in pathologic BPH tissues had not been previously implicated in BPH or prostate cancer. These genes included *F10* (upregulated 16-fold), *LIPG*, *SGCA* and *SMOC1* (upregulated 26-fold). Coagulation Factor X (*F10*) is a serine protease that can be activated by cancer procoagulant (CP), a cysteine protease produced by malignant and embryonic tissues [61]. In addition to promoting blood coagulation, coagulation proteases induce signal transduction through the activation of G protein–coupled protease-activated receptors (PARs) [62], [63], [64], [65], [66]. Additionally, activated Factor X (Factor Xa) can mediate signal transduction via specific binding to annexin 2 [67]. Endothelial lipase (*LIPG*) is involved in lipoprotein metabolism and is elevated in inflammation [68], [69]. *SGCA* (sarcoglycan alpha) encodes a component of the dystrophin-glycoprotein complex (DGC) in striated muscle, which is critical to the stability of muscle fiber membranes and to the linking of the actin cytoskeleton to the extracellular matrix. [70], [71]. Secreted modular calcium-binding protein 1 (*SMOC1*) belongs to the BM-40 family, which has been implicated in tissue remodeling, angiogenesis and bone mineralization [72]. It is widely expressed in many tissues and is a component of the basement membrane [73]. The potential functional role of these genes, identified as androgen regulated in intact human prostate TZ tissues as a novel observation herein, in the pathologic glandular and stromal hyperplasia characteristic of BPH remains to be elucidated. Their spectrum of biologic and pathophysiologic functions is intriguing and further demonstrates the potential of our investigational approach to uncover unique target genes not identified in simple models of cell lines or non-hormonally manipulated tissues.

Analysis of gene expression differences in RNA samples extracted from intact tissues does not specifically allow for localization of the increased or decreased altered genes to the glandular and/or stromal compartments in TZ tissues, both of which likely contribute fundamentally to evolution of BPH, nor does it necessarily translate to altered levels of more biologically relevant proteins. We examined the expression of a subset of genes of interest in BPH tissues at the protein level by immunohistochemistry (IHC), using a tissue microarray containing sections with BPH pathologies ranging from minimal to severe. IHC staining patterns were assessed semi-quantitatively for FGF2, SMOC1, and TIMP2. The expression of FGF2 in BPH has been previously reported by several groups [74], [75], [76]. This is the first published report describing the expression of SMOC1 and TIMP2 in BPH tissue.

As all three of these genes have been found to have altered expression in prostate cancer tissues and in much larger volumes of BPH tissue (by qRT-PCR), they may very well play a role in BPH progression, but based on results of IHC, it does not appear to be simply based on uniform over-expression throughout the transition zone.

The fact that expression arrays on intact human TZ tissues xenografted into hormonally manipulated host mice identified many genes well known to be androgen regulated validates our approach. However, this method may still miss genes with low expression levels that could be important in pathogenesis, although such genes would seem less likely to make good biomarkers. When we examined the expression of a subset of these highly expressed genes from the microarray data by RT-PCR in BPH tissues, we found several with elevated levels in BPH tissues relative to normal controls. Our study confirms the known or postulated role of many of these in prostate pathology, and identifies additional genes that appear worthy of further investigation. The detailed analysis of each of these genes will require substantial further investigation, but may provide further insight into the molecular etiology of BPH pathogenesis.

In summary, our results demonstrate the feasibility of using a mouse xenograft model to characterize the gene expression profiles of intact human prostate tissues in response to androgens. The model has the unique advantage of maintaining intact epithelial-stromal interactions during hormone manipulation, cellular interactions which are likely crucial in the progressive evolution of this androgen dependent pathologic condition. A number of genes were identified that were regulated by androgens, some that were previously well characterized, and many more whose possible roles in prostate development and pathology remain largely unknown. Additionally, a subset of these androgen regulated genes were found to be over-expressed in RNA from clinical BPH tissues, and the levels of many were found to correlate with disease severity. Such knowledge may aid the discovery of new diagnostic and therapeutic targets for the early detection and treatment of BPH and prostate cancer.
